# Adjuvant chemotherapy may improve long-term outcomes in stage IB non-small cell lung cancer patients with previous malignancies: A propensity score-matched analysis

**DOI:** 10.3389/fonc.2022.938195

**Published:** 2022-08-31

**Authors:** Ke Zhou, Yaqin Zhao, Linchuan Liang, Jie Cao, Huahang Lin, Zhiyu Peng, Jiandong Mei

**Affiliations:** ^1^ Department of Thoracic Surgery, West China Hospital, Sichuan University, Chengdu, China; ^2^ Western China Collaborative Innovation Center for Early Diagnosis and Multidisciplinary Therapy of Lung Cancer, Sichuan University, Chengdu, China; ^3^ Cancer Center of West China Hospital, Sichuan University, Chengdu, China

**Keywords:** chemotherapy, stage IB, non-small cell lung cancer, previous malignancy, SEER

## Abstract

**Background:**

Routine administration of adjuvant chemotherapy for stage IB non-small cell lung cancer (NSCLC) remains controversial. To our knowledge, no available studies have assessed the outcomes of chemotherapy in patients with stage IB NSCLC who had prior malignancies.

**Methods:**

Patients with pathological stage IB NSCLC with previous malignancies who underwent surgery between 2004 and 2015 were identified from the Surveillance, Epidemiology, and End Results (SEER) database. The patients were categorized into chemotherapy and observation group based on whether they received adjuvant chemotherapy. Propensity score matching was performed to reduce confounding bias, and Kaplan–Meier curves and log-rank tests were used to compare overall survival (OS) and cancer-specific survival (CSS) between the two groups. Subgroup analyses of the matched cohorts were then conducted to evaluate the relationship between clinical features and chemotherapy.

**Results:**

A total of 894 eligible patients were identified; 90 (10.1%) patients received postoperative chemotherapy. Patients who received adjuvant chemotherapy obtained obvious OS benefits compared with those who received observation alone (HR = 0.68, 95% CI: 0.48–0.97, *P* = 0.031). In addition, the 5-year OS rate and median OS time in the chemotherapy group were higher and longer, respectively. Although chemotherapy offered no obvious benefits for CSS (HR = 0.80, 95% CI: 0.57–1.14, *P* = 0.35), patients who received chemotherapy showed a better 5-year CSS rate. On subgroup analyses, a chemotherapy advantage was observed in advanced age (≥65 years, HR = 0.62, 95% CI: 0.38–0.99, *P* = 0.045). The same chemotherapy advantages were observed in patients diagnosed with higher histological grades (poorly differentiated to undifferentiated) (HR = 0.56, 95% CI: 0.33–0.96, *P* = 0.033) and tumor sizes >3.1–4 cm (HR = 0.57, 95% CI: 0.37–0.87, *P* = 0.010). Interestingly, NSCLC patients with previous malignancies originating from the kidney and bladder (HR = 0.34, 95% CI: 0.12–0.99, *P* = 0.049) showed a chemotherapy advantage. The same chemotherapy advantages were observed in patients diagnosed with NSCLC within 3 to 5 years after prior cancers (HR = 0.39, 95% CI: 0.16–0.98, *P* = 0.044) and with localized SEER stage of prior cancers (HR = 0.49, 95% CI: 0.29–0.86, *P* = 0.012).

**Conclusion:**

These findings indicate that adjuvant chemotherapy may improve long-term outcomes for stage IB NSCLC patients with previous malignancies. It is recommended that physicians consider the clinical features of previous cancers when making adjuvant chemotherapy decisions for these patients.

## Introduction

Lung cancer is prevalent worldwide and has a high risk of morbidity and mortality (1). Non-small cell lung cancer (NSCLC) accounts for 85% of all diagnosed lung cancers, and adenocarcinoma and squamous cell carcinoma are the most common histological subtypes (2). In general, a previous cancer diagnosed within 5 years of the current one is one of the most common exclusion criteria across clinical trials (3). However, an estimated 21% of early-stage lung cancer patients have had prior cancers (4). In general, stage IB NSCLC patients have a 5-year overall survival (OS) rate of 73% (5); in early-stage patients, a history of prior cancer adversely affects OS (6).

Many patients suffer from postoperative recurrence due to the heterogeneous prognosis of stage IB disease. Postoperative adjuvant chemotherapy can reduce the onset of recurrence and metastasis in NSCLC (7). Therefore, adjuvant chemotherapy is recommended after radical resection in patients with risk factors; these include poorly differentiated tumors, vascular invasion, visceral pleural invasion, unknown lymph node status, and wedge resection (8). However, routine administration of adjuvant therapy remains controversial, even in NSCLC patients with risk factors (9, 10). Some studies suggest that adjuvant chemotherapy has beneficial effects (11–14), whereas others advise against its use (15, 16).

Many studies have discussed the impact of adjuvant chemotherapy among patients without previous malignancies. However, it remains unclear whether adjuvant chemotherapy may improve overall survival in patients with previous malignancy. To our knowledge, no available studies have assessed the outcomes of chemotherapy in patients with previous malignancies. Therefore, this study used the Surveillance, Epidemiology, and End Results (SEER) database to assess the effect of postoperative chemotherapy in patients with stage IB NSCLC who had previous malignancies.

## Materials and methods

### Database and patient selection

Patients diagnosed with NSCLC between 2004 and 2015 were selected from the SEER database (Incidence-SEER Research Plus Data,18 Registries, 2020 Sub [2000–2018]) using the SEER*Stat software (version 8.3.9). The “Site Recode ICD-O-3/WHO 2008 classification” and “Histology type ICD-O-3” variables were used for NSCLC patient selection. The inclusion criteria were as follows: 1) T2aN0M0 stage tumors according to the 8th edition TNM staging system for NSCLC [tumor size >30 and ≤40 mm or tumor size ≤30 mm with visceral pleural invasion (VPI)], and 2) history of only one previous malignancy. The exclusion criteria were as follows: 1) younger than 20 years at diagnosis, 2) diagnosed by autopsy or as per the death certificate, 3) incomplete survival data and follow-up information, 4) surgery not performed, 5) receipt of adjuvant radiotherapy, 6) interval time (defined as the time between the previous cancers and the NSCLC diagnosis) of above 2 months (to exclude synchronous primary cancers), and 7) missing data regarding the previous malignancies. Finally, a total of 894 eligible patients were included in this study. Data regarding patient demographics (year of diagnosis, age, race, sex, and marital status), features of tumors (size, VPI, site, differentiation grade, and pathological subtypes), treatment details (surgical type, number of removed lymph nodes, and history of chemotherapy), and follow-up details (survival status and survival time) were extracted from the SEER database. Previous cancer was characterized by the interval time, type, SEER stage, and any history of adjuvant therapy.

### Statistical analysis

Patients were categorized into a chemotherapy group and an observation group based on whether they received adjuvant chemotherapy. The primary endpoints of our analyses were overall survival (OS) and cancer-specific survival (CSS). OS was defined as the time from diagnosis to either death or last follow-up, while CSS was defined as the time from diagnosis to death from NSCLC or last follow-up. Descriptive statistics were utilized to summarize the demographic and clinical factors of these two groups of patients. Clinicopathological characteristics were compared between patients in the chemotherapy and observation groups using the chi-square or Fisher’s exact tests. Propensity score matching (PSM) was then employed to reduce the confounding bias in baseline characteristics, including the year of diagnosis, sex, age, site, ethnicity, pathological grade, histological type, tumor size, VPI, surgical methods, and clinical features of previous malignancies. PSM was performed in a ratio of 1:4 and a caliper of 0.2. Kaplan–Meier plots and log-rank tests were used to compare the OS between the two groups. In the matched cohort, a multivariate Cox proportional hazards regression model (Cox model) was used to estimate hazard ratios (HRs) in the subgroup analysis for the association between variables and chemotherapy effects. Descriptive statistics, chi-square tests, Fisher’s exact tests, construction of Kaplan–Meier plots, log-rank tests, PSM, and Cox regression were all performed using R software version 4.0.5. A two-sided *P*-value of <0.05 was considered to be statistically significant.

## Results

### Baseline characteristics

**Figure 1:**
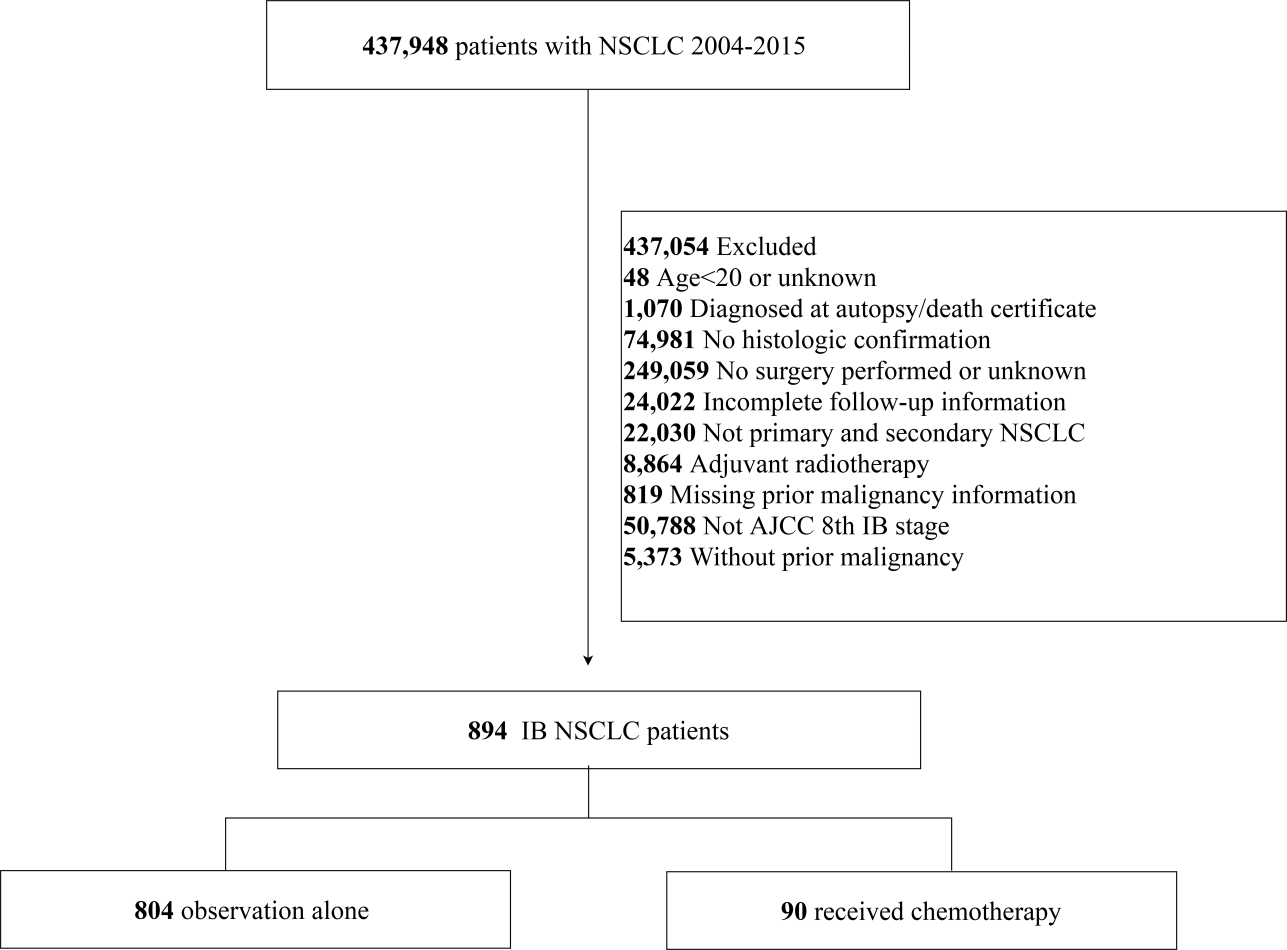
Flowchart of patient selection from the SEER database.

A total of 894 patients with stage IB NSCLC who had a prior malignancy and underwent surgery between 2004 and 2015 were included in this study (**Figure 1**). The NSCLC cases comprised 60.9% of adenocarcinoma, 32.2% of squamous cell carcinoma, 1.45% of large cell carcinoma, and 5.48% of other histologies. There were 523 men and 371 women; 78.1% were more than 65 years of age. The number of cases increased in 2012–2015 (52.1%), compared with 2008–2011 (33.6%) and 2004–2007 (14.3%) (**Table 1**).

**Table 1:** Baseline characteristics of stage IB NSCLC patients with previous malignancies in the SEER database cohort.

		Original dataset			Matched dataset		
Characteristic	Total	Observation	Chemotherapy	*P*-value	Observation	Chemotherapy	*P*-value
Number	894	804	90		335	88	
Clinical features							
Age				0.001*			0.726
<65	196 (21.9%)	163 (20.3%)	33 (36.7%)		109 (32.5%)	31 (35.2%)	
≥65	698 (78.1%)	641 (79.7%)	57 (63.3%)		226 (67.5%)	57 (64.8%)	
Sex				0.795			0.877
Female	371 (41.5%)	332 (41.3%)	39 (43.3%)		143 (42.7%)	39 (44.3%)	
Male	523 (58.5%)	472 (58.7%)	51 (56.7%)		192 (57.3%)	49 (55.7%)	
Race				0.936			0.906
White	741 (82.9%)	667 (83.0%)	74 (82.2%)		269 (80.3%)	72 (81.8%)	
Black	101 (11.3%)	91 (11.3%)	10 (11.1%)		44 (13.1%)	10 (11.4%)	
Other	52 (5.82%)	46 (5.72%)	6 (6.67%)		22 (6.57%)	6 (6.82%)	
Marital status				0.263			0.25
Married	526 (58.8%)	470 (58.5%)	56 (62.2%)		187 (55.8%)	55 (62.5%)	
Ever married	247 (27.6%)	228 (28.4%)	19 (21.1%)		94 (28.1%)	19 (21.6%)	
Never married	81 (9.06%)	69 (8.58%)	12 (13.3%)		34 (10.1%)	12 (13.6%)	
Unknown	40 (4.47%)	37 (4.60%)	3 (3.33%)		20 (5.97%)	2 (2.27%)	
Site				0.419			0.255
Main bronchus	1 (0.11%)	1 (0.12%)	0 (0.00%)		1 (0.30%)	0 (0.00%)	
Upper lobe	521 (58.3%)	464 (57.7%)	57 (63.3%)		189 (56.4%)	55 (62.5%)	
Middle lobe	53 (5.93%)	50 (6.22%)	3 (3.33%)		27 (8.06%)	3 (3.41%)	
Lower lobe	306 (34.2%)	278 (34.6%)	28 (31.1%)		114 (34.0%)	28 (31.8%)	
Overlapping lesion of the lung	10 (1.12%)	9 (1.12%)	1 (1.11%)		4 (1.19%)	1 (1.14%)	
Lung, NOS	3 (0.34%)	2 (0.25%)	1 (1.11%)		0 (0.00%)	1 (1.14%)	
Laterality				0.813			0.877
Left	372 (41.6%)	333 (41.4%)	39 (43.3%)		143 (42.7%)	39 (44.3%)	
Right	522 (58.4%)	471 (58.6%)	51 (56.7%)		192 (57.3%)	49 (55.7%)	
Grade				0.068			0.874
G1	121 (13.5%)	113 (14.1%)	8 (8.89%)		31 (9.25%)	8 (9.09%)	
G2	411 (46.0%)	373 (46.4%)	38 (42.2%)		151 (45.1%)	38 (43.2%)	
G3	300 (33.6%)	261 (32.5%)	39 (43.3%)		143 (42.7%)	38 (43.2%)	
G4	7 (0.78%)	5 (0.62%)	2 (2.22%)		4 (1.19%)	1 (1.14%)	
Unknown	55 (6.15%)	52 (6.47%)	3 (3.33%)		6 (1.79%)	3 (3.41%)	
Histology				0.822			0.936
ADC	544 (60.9%)	491 (61.1%)	53 (58.9%)		201 (60.0%)	52 (59.1%)	
SCC	288 (32.2%)	258 (32.1%)	30 (33.3%)		115 (34.3%)	30 (34.1%)	
LCC	13 (1.45%)	11 (1.37%)	2 (2.22%)		7 (2.09%)	2 (2.27%)	
Other	49 (5.48%)	44 (5.47%)	5 (5.56%)		12 (3.58%)	4 (4.55%)	
Tumor size, cm				0.641			0.982
≤2	205 (22.9%)	187 (23.3%)	18 (20.0%)		67 (20.0%)	18 (20.5%)	
2.1–3	154 (17.2%)	140 (17.4%)	14 (15.6%)		56 (16.7%)	14 (15.9%)	
3.1–4	535 (59.8%)	477 (59.3%)	58 (64.4%)		212 (63.3%)	56 (63.6%)	
VPI				0.267			0.805
No	462 (51.7%)	410 (51.0%)	52 (57.8%)		183 (54.6%)	50 (56.8%)	
Yes	432 (48.3%)	394 (49.0%)	38 (42.2%)		152 (45.4%)	38 (43.2%)	
LNs, *n*				0.724			0.159
0	114 (12.8%)	101 (12.6%)	13 (14.4%)		41 (12.2%)	13 (14.8%)	
≤10	478 (53.5%)	432 (53.7%)	46 (51.1%)		201 (60.0%)	44 (50.0%)	
>10	232 (26.0%)	206 (25.6%)	26 (28.9%)		66 (19.7%)	26 (29.5%)	
Unknown	70 (7.83%)	65 (8.08%)	5 (5.56%)		27 (8.06%)	5 (5.68%)	
Surgery				0.703			0.82
Lobectomy	654 (73.2%)	589 (73.3%)	65 (72.2%)		238 (71.5%)	63 (71.6%)	
Sublobar resection	224 (25.1%)	201 (25.0%)	23 (25.6%)		90 (27.0%)	23 (26.1%)	
Pneumonectomy	11 (1.23%)	9 (1.12%)	2 (2.22%)		5 (1.50%)	2 (2.27%)	
Other	5 (0.56%)	5 (0.62%)	0 (0.00%)				
Year at diagnosis				0.078			0.138
2004–2007	128 (14.3%)	108 (13.4%)	20 (22.2%)		45 (13.4%)	19 (21.6%)	
2008–2011	300 (33.6%)	273 (34.0%)	27 (30.0%)		121 (36.1%)	26 (29.5%)	
2012–2015	466 (52.1%)	423 (52.6%)	43 (47.8%)		169 (50.4%)	43 (48.9%)	
Prior malignancy							
Interval time, years				0.499			0.932
<1	172 (19.2%)	154 (19.2%)	18 (20.0%)		61 (18.2%)	17 (19.3%)	
1–3	219 (24.5%)	193 (24.0%)	26 (28.9%)		87 (26.0%)	25 (28.4%)	
3–5	161 (18.0%)	143 (17.8%)	18 (20.0%)		69 (20.6%)	18 (20.5%)	
>5	342 (38.3%)	314 (39.1%)	28 (31.1%)		118 (35.2%)	28 (31.8%)	
Types				0.378			0.989
Lung and bronchus	125 (14.0%)	108 (13.4%)	17 (18.9%)		59 (17.6%)	17 (19.3%)	
Breast	121 (13.5%)	111 (13.8%)	10 (11.1%)		38 (11.3%)	10 (11.4%)	
Head and neck	77 (8.61%)	70 (8.71%)	7 (7.78%)		30 (8.96%)	6 (6.82%)	
Digestive system	139 (15.5%)	131 (16.3%)	8 (8.89%)		40 (11.9%)	8 (9.09%)	
Blood system	56 (6.26%)	47 (5.85%)	9 (10.0%)		32 (9.55%)	8 (9.09%)	
Kidney and bladder	104 (11.6%)	91 (11.3%)	13 (14.4%)		51 (15.2%)	13 (14.8%)	
Female reproductive system	32 (3.58%)	28 (3.48%)	4 (4.44%)		11 (3.28%)	4 (4.55%)	
Male reproductive system	173 (19.4%)	158 (19.7%)	15 (16.7%)		48 (14.3%)	15 (17.0%)	
Other	67 (7.49%)	60 (7.46%)	7 (7.78%)		26 (7.76%)	7 (7.95%)	
SEER stages				0.87			0.72
Localized	406 (45.4%)	364 (45.3%)	42 (46.7%)		158 (47.2%)	42 (47.7%)	
Regional	356 (39.8%)	323 (40.2%)	33 (36.7%)		110 (32.8%)	33 (37.5%)	
Distant	96 (10.7%)	85 (10.6%)	11 (12.2%)		48 (14.3%)	9 (10.2%)	
Unknown	36 (4.03%)	32 (3.98%)	4 (4.44%)		19 (5.67%)	4 (4.55%)	
Ever received adjuvant therapies				0.923			0.76
No	496 (55.5%)	447 (55.6%)	49 (54.4%)		195 (58.2%)	49 (55.7%)	
Yes	398 (44.5%)	357 (44.4%)	41 (45.6%)		140 (41.8%)	39 (44.3%)	

Ever married included widowed and separated.

G1, well-differentiated; G2, moderately differentiated; G3, poorly differentiated; G4, undifferentiated; ADC, adenocarcinoma; SCC, squamous cell carcinoma; LCC, large cell carcinoma; VPI, visceral pleural invasion; LNs, lymph node numbers.

*P-values < 0.05.

Overall, the most common tumor types were from the male reproductive system (19.4%), digestive system (15.5%), and lung and bronchus (14%); 61.7% of patients were identified as having NSCLC within 5 years after prior cancers. The median interval between the diagnosis of NSCLC and their previous malignancies was 45 months (range: 2–185 months); according to the SEER stage, 45.4% of patients were diagnosed with previous localized malignancies. In addition, 44.5% of patients were recognized to have received corresponding adjuvant therapies for previous cancers (**Table 1**).

In the entire population, 90 patients were recognized to have received adjuvant chemotherapy after surgery. Among the 90 patients who underwent surgery, 65 and 23 underwent lobectomy and sublobar resection, respectively; 57 patients were older than 65 years and 51 patients were men (**Table 1**). In terms of the clinical features of previous malignancies, patients diagnosed with NSCLC within 1–3 years after prior malignancies and those with localized previous cancers were more likely to receive chemotherapy (12%). The same results were observed in patients with previous tumors originating from the blood system (16%), lung and bronchus (14%), and kidney and bladder (13%).

### Survival analysis

**Figure 2:**
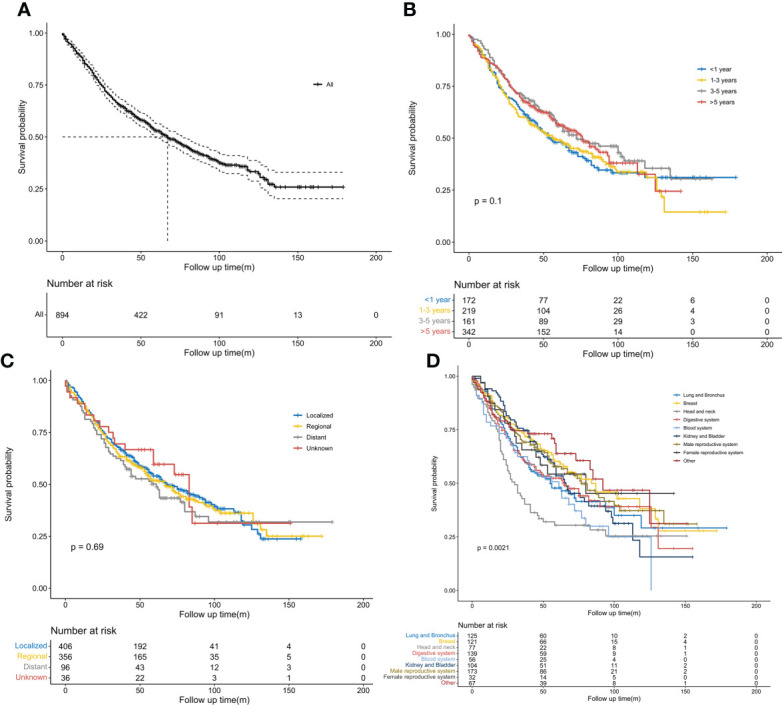
Kaplan–Meier survival curves. **(A)** Overall survival probability in patients with stage IB non-small cell lung cancer (NSCLC) having previous malignancies. **(B)** Comparison of overall survival probability in patients with stage IB NSCLC by interval time after previous cancers. **(C)** Comparison of overall survival probability in patients with stage IB NSCLC by SEER stages of previous malignancies. **(D)** Comparison of overall survival probability in patients with stage IB NSCLC by types of previous malignancies.

The Kaplan–Meier curves for OS are shown in **Figure 2**. The 5-year OS rate of the entire population was 53.0%, while the median OS was 67 months (**Figure 2A**). No significant survival difference was observed on stratification by interval time (*P* > 0.05). However, the 5-year OS rate was the highest in the above 5-year group (56.7%) and the lowest in the within 1-year group (48%) (**Figure 2B**). The median OS was 76 months in the above 5-year group and 55 months in the within 1-year group. No obvious significant survival difference was observed on stratification by SEER stages of the previous cancers (*P* > 0.05). However, localized stages were associated with a higher 5-year OS rate of 54%, while distant metastases conferred the lowest rate of 50.1% (**Figure 2C**). The median OS times for patients with distant metastases and localized disease were 61 and 68 months, respectively. An obvious significant survival difference was observed on stratification by type of previous cancers (*P* < 0.05). NSCLC patients with previous head and neck tumors achieved the poorest survival, with a 5-year OS rate of 30.44% and a median OS of 30 months; this was followed by lung and bronchus cancers, with a 5-year OS rate of 47.8% and a median OS of 56 months (**Figure 2D**).

**Figure 3:**
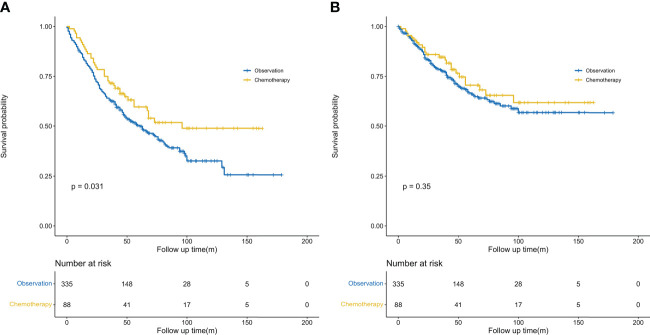
Kaplan–Meier survival curves. **(A)** Comparison of overall survival probability (after PSM) by treatment method in patients with stage IB NSCLC having previous cancers. **(B)** Comparison of cancer-specific survival probability (after PSM) by treatment method in patients with stage IB NSCLC having previous cancers. PSM, propensity score matching.

We further conducted PSM analysis between the chemotherapy and observation-alone groups based on the following variables: year of diagnosis, sex, age, site, ethnicity, pathological grade, histological type, tumor size, VPI, surgical methods, and clinical features of previous malignancies. Finally, 88 patients who received chemotherapy were matched with 335 patients who did not receive chemotherapy (1:4). The clinical characteristics did not differ significantly between the two groups of patients (**Table 1**). As evidenced by the Kaplan–Meier curves and the results of the log-rank analysis for OS, chemotherapy conferred obvious survival benefits (HR = 0.68, 95% CI: 0.48–0.97, *P* = 0.031) (**Figure 3A**). The 5-year OS rates in the chemotherapy and observation groups were 59.7% and 50.3%, respectively. The median OS was 96 months in the chemotherapy group; this was longer than that in the observation group (61 months). Although chemotherapy conferred no obvious benefits on CSS (HR = 0.80, 95% CI: 0.57–1.14, *P* = 0.35) (**Figure 3B**), it offered better CSS tendency, with a 5-year CSS rate of 70.6%; this was higher than the rate of 66.7% observed in the observation group.

### Subgroup analyses

**Figure 4:**
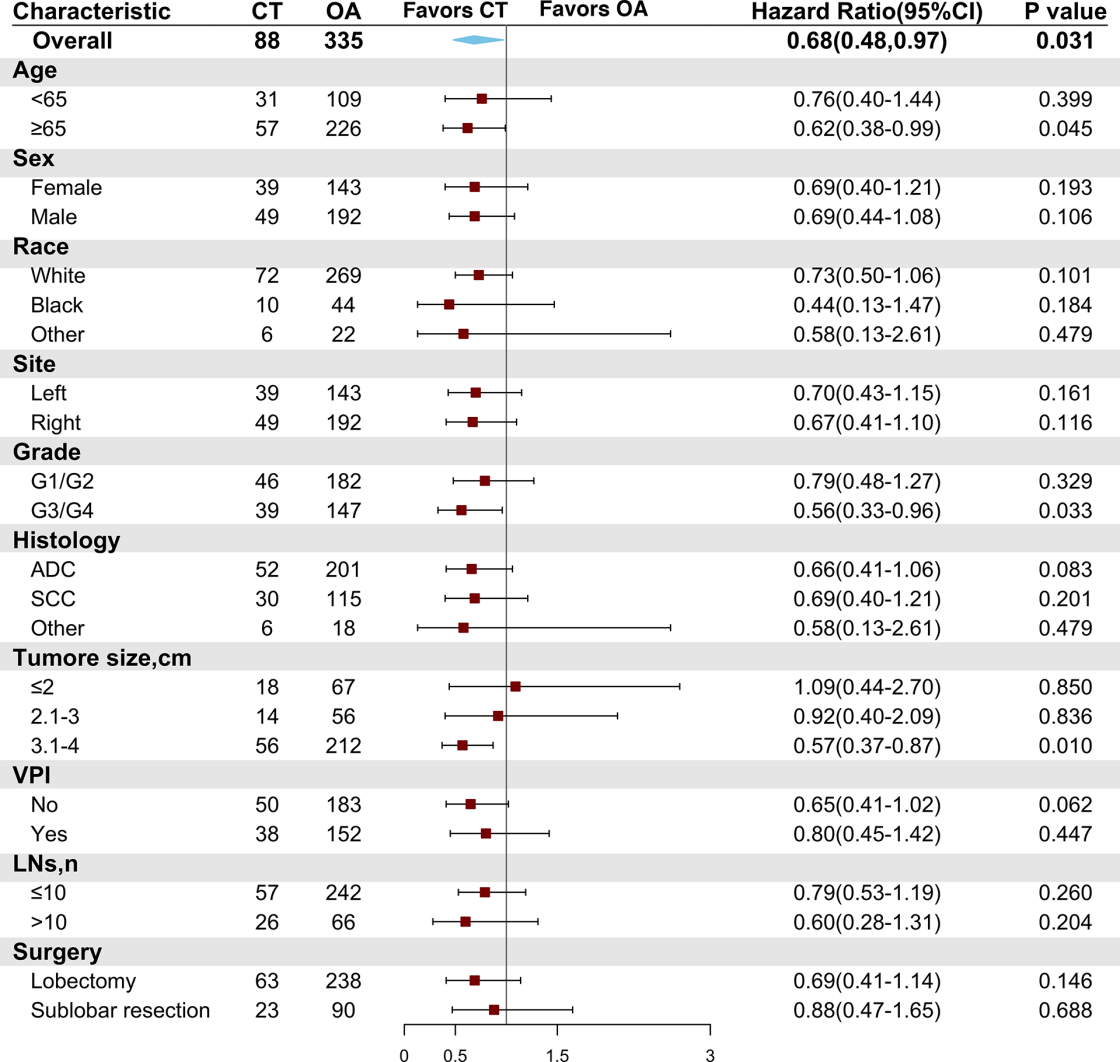
Comparison of overall survival between the chemotherapy and observation groups in the matched cohort according to patient clinical characteristics. CT, chemotherapy; OA, observation alone; G1, well-differentiated; G2, moderately differentiated; G3, poorly differentiated; G4, undifferentiated; ADC, adenocarcinoma; SCC, squamous cell carcinoma; VPI, visceral pleural invasion; LNs, lymph node numbers.

Subgroup analyses were conducted in the PSM cohort using the Cox model to evaluate the associations between a high risk of clinical features and adjuvant chemotherapy. A chemotherapy advantage was observed in patients aged ≥65 years (HR = 0.62, 95% CI: 0.38–0.99, *P* = 0.045). The same chemotherapy advantages were observed in patients diagnosed with tumors of higher histological grade (poorly differentiated to undifferentiated grade) (HR = 0.56, 95% CI: 0.33–0.96, *P* = 0.033) and tumor size ranging from 3.1 to 4 cm (HR = 0.57, 95% CI: 0.37–0.87, *P* = 0.01). However, patients diagnosed with VPI did not obtain superior benefit from chemotherapy (HR = 0.80, 95% CI: 0.45–1.42, *P* = 0.447). In addition, neither lobectomy (HR = 0.69, 95% CI: 0.41–1.14, *P* = 0.146) nor sublobar resection (HR = 0.88, 95% CI: 0.47–1.65, *P* = 0.688) conferred better survival after chemotherapy (**Figure 4**).

**Figure 5:**
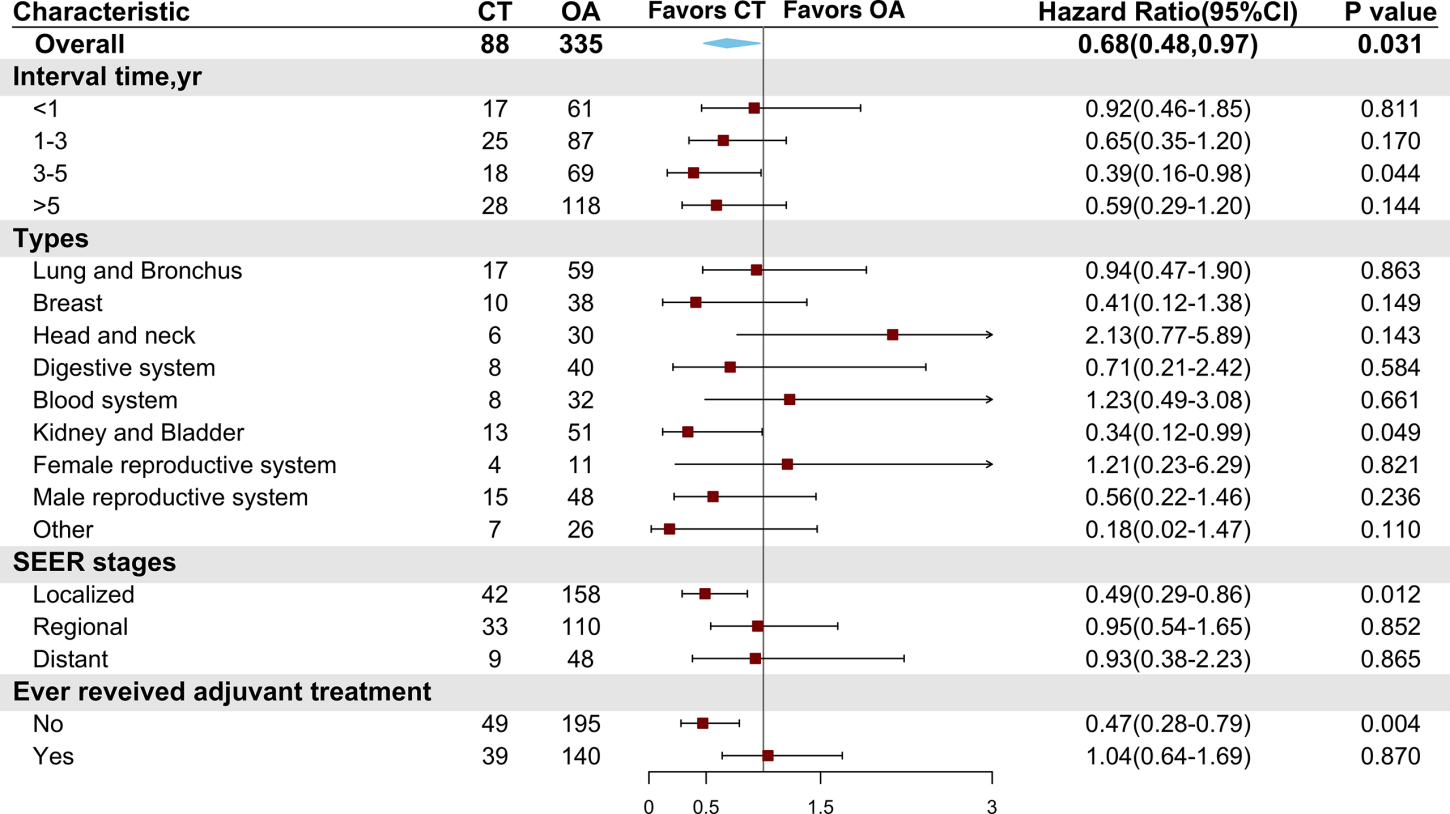
Comparison of overall survival between the chemotherapy and observation groups in the matched cohort according to characteristics of previous malignancies. CT, chemotherapy; OA, observation alone.

As NSCLC patients with different previous malignancies showed variable willingness to receive adjuvant chemotherapy, we also conducted subgroup analyses to explore the effect of chemotherapy on patients with different clinical features of previous malignancies. We found that NSCLC with malignancies of the kidney and bladder (HR = 0.34, 95% CI: 0.12–0.99, *P* = 0.049) showed a chemotherapy advantage. The same chemotherapy advantages were observed in patients diagnosed with NSCLC within 3 to 5 years after previous malignancies (HR = 0.39, 95% CI: 0.16–0.98, *P* = 0.044) and in those with a localized SEER stage of prior cancers (HR = 0.49, 95% CI: 0.29–0.86, *P* = 0.012). Interestingly, those who had not received adjuvant therapies for previous malignancies (HR = 0.47, 95% CI: 0.28–0.79, *P* = 0.004) had better OS than those being administered with chemotherapy (**Figure 5**).

## Discussion

As the prognosis of stage IB NSCLC differs remarkably, the use of routine adjuvant chemotherapy after radical surgery in driver mutation-negative patients is debated (9, 10). According to the National Comprehensive Cancer Network guidelines (17), adjuvant chemotherapy is recommended for patients with stage IB NSCLC with high-risk factors; these include poorly differentiated tumors, vascular invasion, VPI, unknown lymph node status, and wedge resection. Many studies have discussed the impact of adjuvant chemotherapy for patients with stage IB NSCLC without any previous malignancy (11, 12, 14, 16). To our knowledge, the outcomes of chemotherapy have not been discussed in patients with previous malignancies.

Our study showed that patients with previous malignancies achieved long-term benefit from chemotherapy after surgery. In addition, those receiving chemotherapy showed a longer median OS and a higher 5-year OS rate than patients in the observation group. Although chemotherapy did not improve CSS in patients receiving postoperative chemotherapy, they achieved a higher 5-year CSS rate. This may be attributed to the fact that relapse of previous tumors may adversely affect long-term prognosis. Therefore, chemotherapy may offer benefits in terms of long-term prognosis in patients with stage IB NSCLC who have a history of malignancy; this may be achieved by removal of recurrence risk for previous and current cancers. On subgroup analyses, we found that patients with advanced age (≥65 years) could benefit from chemotherapy. Previous studies showed that age did not impact the effect of chemotherapy in patients with NSCLC, and even patients older than 70 years could benefit from adjuvant chemotherapy (18, 19). Prior studies have shown that VPI and poorly differentiated tumors confer a higher risk of recurrence and death after surgical resection (16, 20, 21). Chemotherapy, as an effective treatment, may contribute to the removal of residual lesions. However, tumors with VPI did not show a chemotherapy advantage; this finding agreed with that of a previous study (16). On the contrary, patients with poorly differentiated to undifferentiated tumors achieved better OS from adjuvant chemotherapy in our study. Multiple studies have sought to explore the association between tumor size and chemotherapy. Reports indicate that adjuvant chemotherapy offers benefits in tumors larger than 3 cm, including those measuring 3.1 to 3.9 cm (10). Our study also demonstrated a chemotherapy benefit for tumors larger than 3.1 to 4 cm. A previous study suggested that chemotherapy could remove the residual cancer risk after sublobar resection (including wedge resection and segmentectomy) (22). However, neither sublobar resection nor lobectomy was related to chemotherapy benefit in our study; this concurs with the findings of a previous study (23). A survey indicated that inadequate lymph node sampling may lead to incorrect stage evaluation and chemotherapy could eliminate the cancer risk for these patients (24, 25). However, we found no chemotherapy advantage in patients with fewer than 10 examined nodes.

Pruitt et al. (4) claimed that 21% of patients with early-stage lung cancer had prior cancers. The most common prior cancers were prostate, breast, gastrointestinal, and other genitourinary cancers. Most prior cancers were localized, and 61% were diagnosed within 5 years of the lung cancer diagnosis. Our study showed similar findings in that over half of the patients developed NSCLC within 5 years after prior cancers and most prior cancers were localized. However, the most common types of previous malignancies originated from the male reproductive system (mainly the prostate), digestive system, and lung and bronchus; active surveillance for NSCLC is therefore recommended for survivors of these cancers. In this context, we found that nearly half of NSCLC patients with previous malignancies received corresponding adjuvant therapies in the past.

The OS, stratified in terms of features of previous malignancies, did not show any differences except for between tumor types; however, the median OS and 5-year survival rate were prolonged with an increase in interval time. Interestingly, NSCLC patients with different previous malignancies showed variable willingness to receive adjuvant chemotherapy. Those with previous tumors of the blood system, kidney and bladder, and lung and bronchus were more likely to choose adjuvant chemotherapy. We therefore conducted subgroup analyses to explore the effect of chemotherapy on patients with different clinical features of previous malignancies. We observed that those with previous tumors originating from the kidney and bladder achieved significant chemotherapy benefit. However, those with prior cancers of the blood system and lung and bronchus did not obtain chemotherapy advantage. Interestingly, although NSCLC patients with previous head and neck tumors had poorer OS, chemotherapy offered no OS benefits in these patients. In this context, those with a localized stage of previous cancers or diagnosed with NSCLC within 3 to 5 years after a previous cancer showed superior chemotherapy benefit. Patients receiving no adjuvant treatment for previous cancer were also found to obtain superior chemotherapy benefit. Chemotherapy resistance is prevalent among patients with NSCLC (26); this may be attributed to previous adjuvant therapies and may explain why clinical trials exclude patients with previous cancer. In the context of a clinical trial, previous treatment may affect the outcomes of treatment for lung cancer. The underlying relationship between the characteristics of previous cancers and chemotherapy advantage warrants further research.

Our study has several limitations. First, on account of the absence of data pertaining to vascular space invasion (an indicator for chemotherapy benefit, as described by the National Comprehensive Cancer Network guidelines) and disease-free survival (as an important indicator for evaluating the efficacy of chemotherapy) in the SEER database, we could not comprehensively evaluate the effect of chemotherapy. Furthermore, details of the chemotherapy regimens were not recorded; we could not therefore identify the chemotherapeutic agents that would benefit these patients. In addition, adjuvant therapies for stage IB NSCLC currently consist of immunotherapy, targeted therapy, and chemotherapy. Some patients may receive chemotherapy along with targeted therapy or immunotherapy. However, the lack of data for other therapies may have contributed to inevitable bias for outcomes of chemotherapy. Lastly, selection bias was inevitable on account of strict selection. Considering the deficiency of retrospective analysis, further prospective analysis is warranted.

## Conclusion

The findings from this study indicate that adjuvant chemotherapy may improve long-term outcomes for stage IB NSCLC patients with previous malignancies. It is suggested that physicians consider the clinical features of previous cancers when making decisions for adjuvant chemotherapy in these patients. Further prospective trials are needed to confirm these findings.

## Data availability statement

The datasets presented in this study can be found in online repositories. The names of the repository/repositories and accession number(s) can be found below: SEER database.

## Ethics statement

Ethical approval was not provided for this study on human participants because All procedures performed in studies involving human participants were in accordance with the ethical standards of the institutional and/or national research committee and with the 1964 Helsinki declaration and its later amendments or comparable ethical standards. For this type of study formal consent is waived. The patients/participants provided their written informed consent to participate in this study.

## Author contributions

Conception and design: JM, YZ, and KZ. Administrative support: JM and YZ. Acquisition of data: JC and HL. Statistical analysis: JM, YZ, KZ, JC, and ZP. Interpretation of the data: all authors. Drafting of the manuscript: HL and KZ. All authors reviewed and approved the final version of the manuscript. All authors contributed to the article and approved the submitted version.

## Funding

This work was supported by a grant from the 1.3.5 Project for Disciplines of Excellence (ZYJC18009), West China Hospital, Sichuan University to JM. This study was also supported by the Special Fund for Early Diagnosis and Treatment of Lung Cancer (7083501001001), Sichuan University Education Foundation awarded to YZ.

## Acknowledgments

We would like to thank all the staff of the National Cancer Institute for their efforts toward the SEER program.

## Conflict of interest

The authors declare that the research was conducted in the absence of any commercial or financial relationships that could be construed as a potential conflict of interest.

## Publisher’s note

All claims expressed in this article are solely those of the authors and do not necessarily represent those of their affiliated organizations, or those of the publisher, the editors and the reviewers. Any product that may be evaluated in this article, or claim that may be made by its manufacturer, is not guaranteed or endorsed by the publisher.
